# How to catch trends using MeSH terms analysis?

**DOI:** 10.1007/s11192-022-04292-y

**Published:** 2022-02-21

**Authors:** Ekaterina V. Ilgisonis, Mikhail A. Pyatnitskiy, Svetlana N. Tarbeeva, Artem A. Aldushin, Elena A. Ponomarenko

**Affiliations:** 1 Institute of Biomedical Chemistry, Moscow, Russia 119121; 2 A.S. Puchkov Station of Emergency Medical Assistance, Moscow, Russia

**Keywords:** Text-mining, PubMed, MEDLINE, MeSH, Trends, Personalized medicine, Precision medicine, Automatic text analysis

## Abstract

The paper describes a scheme for the comparative analysis of the sets of Pubmed publications. The proposed analysis is based on the comparison of the frequencies of occurrence of keywords—MeSH terms. The purpose of the analysis is to identify MeSH terms that characterize research areas specific to each group of articles, as well as to identify trends—topics on which the number of published works has changed significantly in recent years. The proposed approach was tested by comparing a set of medical publications and a group of articles in the field of personalized medicine. We analyzed about 700 thousand abstracts published in the period 2009–2021 and indexed them with MeSH terms. Topics with increasing research interest have been identified both in the field of medicine in general and specific to personalized medicine. Retrospective analysis of the keywords frequency of occurrence changes has shown the shift of the scientific priorities in this area over the past 10 years. The revealed patterns can be used to predict the relevance and significance of the scientific work direction in the horizon of 3–5 years. The proposed analysis can be scaled in the future for a larger number of groups of publications, as well as adjusted by introducing filters at the stage of sampling (scientific centers, journals, availability of full texts, etc.) or selecting a list of keywords (frequency threshold, use of qualifiers, category of generalizations).

**Supplementary Information:**

The online version contains supplementary material available at 10.1007/s11192-022-04292-y.

## Introduction

Analyzing the text of biomedical papers allows us to detect the relationship between various entities, for example, proteins, genes, diseases, organs, and tissues, as well as to obtain information about patterns in the development and relevance of scientific studies in various subject fields (Hassani et al., 2019). In the field of medicine and life sciences, the search for papers is mainly carried out via the PubMed/MEDLINE database (Agarwala et al., 2018; Fiorini et al., 2017).

A distinctive feature of the PubMed system is Medical Subject Headings (MeSH), a controlled vocabulary of biomedical terms which allows indexing of papers and other documents to improve its search algorithm (Mao & Lu, 2017). Each article is indexed manually by MEDLINE experts with 10–15 MeSH terms. The MeSH dictionary is updated every year (over 30,000 terms in 2020) and is maintained by the National Library of Medicine. The hierarchical structure of the dictionary is organized into 16 main categories, e.g., Anatomy, Diseases, Chemical Compounds, and Analytical Methods.

Since 2001, various add-on tools for PubMed have been actively developed, allowing improvement and modification of search queries in MEDLINE, or visualization of the results. Methods for assessing the subject field through the use of MeSH terms is the basis for many derivatives of PubMed (Lu, 2011; Yang & Lee, 2018), such as GoPubMed (Doms & Schroeder, 2005) and PubMed PubReMiner (Eom & Zhang, 2004). A lot of them were implemented for the research trends revealing and analysis. We will name just some of them further, not to distract the reader from our study, but one can easily find their detailed description in Zhang et al., for example (Zhang et al., 2014).

Balogh et al. examined various statistical properties of the Mesh term networks time evolution, with a special focus on the attachment and detachment mechanisms of the links, and find a few general features that are characteristic for all MeSH hierarchies (Balogh et al., 2019).

Mesh terms frequency occurrence was used in the drug-drug interactions research (Lu et al., 2017).

The co-occurrence heatmaps and networks generated from these MeSH terms were used to explore the drug-drug interactions and also illuminated possible associations among drugs, proteins, and phenomena, which can help people understand DDIs better.

In 2018, Yang and Lee (2018) proposed a prototype for a visualization algorithm of the research field in the form of a network consisting of MeSH terms. The prototype included a mechanism for selecting the most important MeSH terms, and a similar algorithm was used in our study.

The key difference of our algorithm from other existing Pubmed derivatives is that it can be used not only for identifying and visualizing relationships between MeSH descriptors, but to compare two ore more sets of Mesh-terms, indexing high-specialized papers.

The number of papers in Pubmed is growing, and the factors contributing to the overall growth of PubMed records during includes journals, publication types, electronic or print availability, open or subscription access, funding, author affiliation, language and home country of publisher (Vardakas et al., 2015). That’s why in our study we recommend to analyze Mesh occurrence frequencies for target and control groups. We’ve analyzed frequencies of MeSH term occurrence for the publications in the area of personalized medicine, one of the general trends in modern medicine, transitioning from the evidence-based statistical approaches to the high-detail investigation of the individual cases. This is extremely important for the creation of digital copies of the person, disease, and society, and to estimate how various factors affect the behavior of the system.

The proposed algorithm could be considered as a valuable addition to the previously published approaches in the field of the MeSH terms frequency analysis for various applications in the area of scientometric analysis.

The analysis of scientific trends is important from the perspective of investment. Like the stock market, research areas also have their ups and downs, and usage of accurate scientometric approaches will assure the best investment of time, efforts, and resources of the research groups. It is extremely important in the context of shifting towards formal KPIs in the research field, interconnecting the quantity and quality of the publications and research funding.

In the future, we are planning to develop a web-based tool allowing for the complete analysis, including a selection of the research area, trend analysis, filtering results based on journals’ impact factors, institutions, authors, full-text availability, etc.

## Materials and methods

### Description of the samples matching scheme

#### Database

The abstract database MEDLINE (Agarwala et al., 2018; Fiorini et al., 2017) was used. The abstracts and related MeSH-terms were accessed via the PubMed search server using API Scanbious (ScanBIOus.). A relevant PubMed identifier, hereinafter referred to as PMID, was ascribed to each MEDLINE entry matching the query Q(t).

#### MeSH-terms’s frequency vectors

**Fig. 1:**
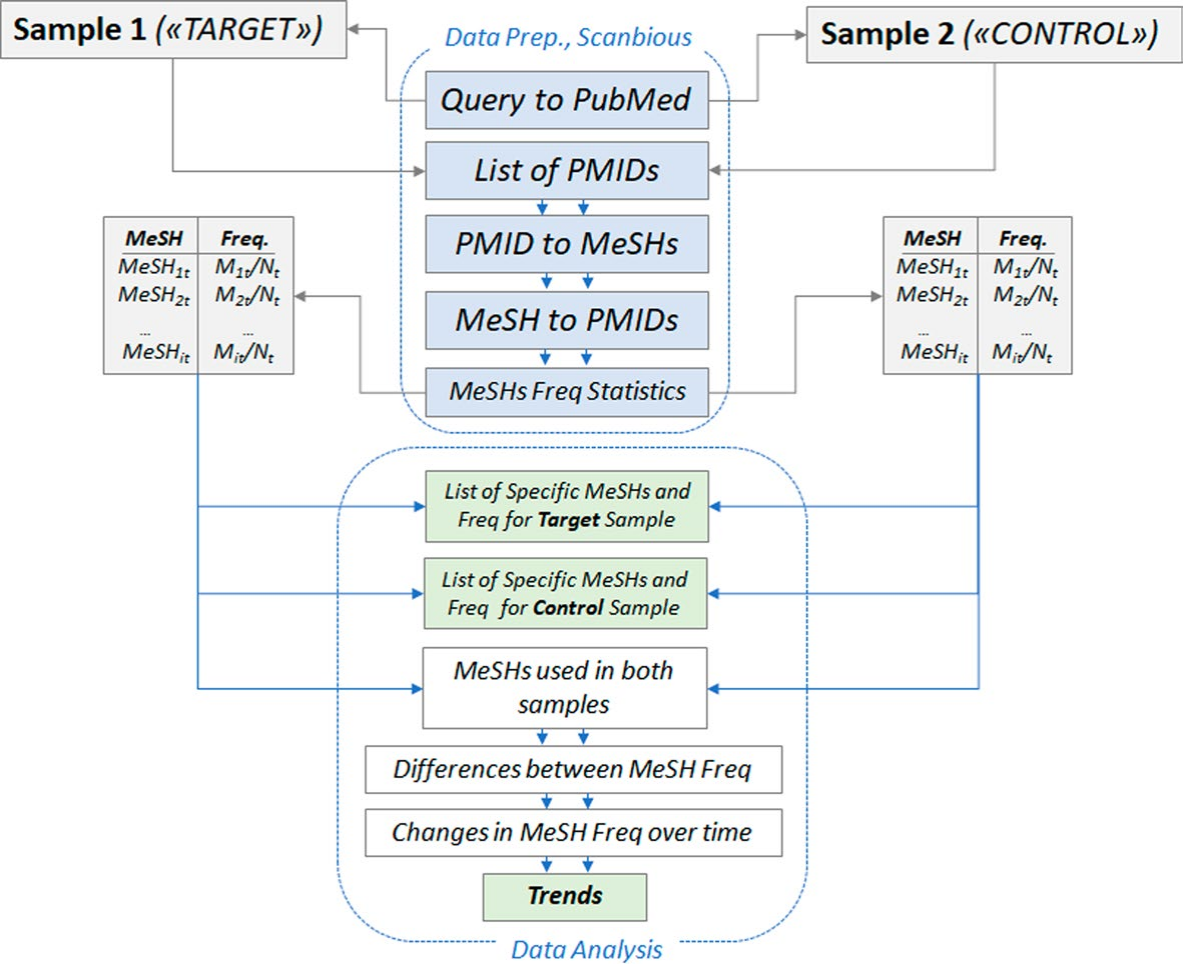
Scheme of comparative analysis of papers related to the different research directions and presented in the form of a target and control sets of papers indexed in the PubMed library using MeSH-terms. N_t_ is the number of articles in the target sample, N_c_ is the number of articles in the control sample. See the description in the text

The sample matching scheme is shown in Fig. 1. It consists of two parts: preparation of samples and input data (Data Preparation) and comparative frequency analysis of keywords—MeSH terms (Frequency vectors analysis). Samples of papers formed based on processing requests to query Q(t) taken into account a target sample (Sample 1 or “*Target*” in Fig. 1) of papers and a background sample (Sample 2 or “*Control*”), in comparison with which the target group of paper is analyzed.

API Scanbious returns a list of PMIDs according to the papers relevant to the Q(t) query to MEDLINE/PubMed (List of PMIDs). Each PMID is associated with a set of MeSH-terms—keywords, characterizing the paper (PMID to MeSHs). Based on these data, Scanbious generates a list of MeSH terms, that characterize each sample, and also calculates the frequency of occurrence of each MeSH-term normalized to the total number of papers in the sample (MeSH-terms’s frequency vectors). Thus, for each sample, a *MeSH-terms’s frequency vector* is formed, which is a set of keywords (MeSH-terms) and its relative frequency of occurrence in a given sample.

#### Data analysis

The relative frequencies of the term used in the samples were compared for each term and year, i.e., the values Mi/Nc and Mj/Nt were computed, where PMi and GMi represent the number of papers annotated by this term from the target and control samples, respectively, and Ni is the total number of papers for the i-th year (equal for both samples). Statistical differences between the frequencies (p-value) were determined using the prop. test function. Correction for multiple comparisons was made using the FDR method (Benjamini & Hochberg, 1995). To obtain a final estimate of the significance of the differences in term use, the obtained set of p-values for each term in 2009–2018 was aggregated using the Fisher method (Fisher, 1925).

In addition to using the p-value to evaluate the significance of the difference in the frequency of use, a ratio ([logratio = log]_2 ([PM]_i/[GM]_i)) characterizing the “magnitude of the effect" for the difference in the occurrence of the term in both samples of papers was calculated for each i-th MeSH term. A positive log ratio value for the MeSH terms indicates that its frequency of occurrence is higher in the target sample compared to the control sample, while a negative value is observed for MeSH terms which are comparatively more often associated with papers on general medicine (control sample). The absolute value of the log ratio indicates the magnitude of this difference. For example, if the log ratio for the term “exercise therapy” for the period 2016–2017 is − 2.0, then this term is four times more likely to be found in the control sample of papers compared to the target sample. If GMi was zero, the log ratio was set as equal to 10.

An analysis of the change in the frequency of occurrence of the MeSH term over time (dynamics and trends) was performed using the nonparametric Mann–Kendall trend test, designed to identify consistently increasing or decreasing trends (Kendall, 1975; Mann, 1945). We used the function MK.test implemented in the trends package (Pohlert, 2020). Graphics were done using word cloud (Fellows, 2018) and ComplexHeatmap (Gu et al., 2016) package.

The dynamics of the frequency of occurrence of MeSH terms of papers for the period 2009–2018 were studied, while MeSH terms for which the data on occurrence were available for less than 6 years were excluded from the analysis. The Mann–Kendall test that was used to identify trends in the frequency of MeSH term usage determines monotonous trends in data that may not necessarily be linear. The Mann–Kendall test is nonparametric (i.e., does not require a normal distribution of data), and analyzes the distribution of the signs of the differences between neighboring values. The determining trend in the dynamics of the term use frequency was considered credible if the *p*-value of the Mann–Kendall test did not exceed the threshold value of 0.01. The correction for multiple comparisons was not applied due to the limited power of the test, since there was a maximum value of 9 for the frequency of each term.

### Test example: input data

Two samples of publications were used as a test example—the target group included articles on personalized medicine (queries of the form [“personalized medicine” [All Fields] OR “precision medicine” [All Fields]]), and the control group included articles on medicine [queries of the form (“medicine” [MeSH])]. The samples were generated for the period 2009–2021 in increments of one year. Statistical information on the samples is presented in Table 1.

**Table 1:** Statistical information on the samples

Date	Personalized medicine (PM)*		General medicine (GM, “control”)**	
	Number of papers indexed with MeSH	Number of MeSH terms	Number of papers with MeSH	Number of MeSH terms
2020–2021	7099	17,641	39,312	36,943
2019–2020	8362	20,626	48,589	46,884
2018–2019	7088	18,244	42,515	47,244
2017–2018	5638	13,535	39,242	37,445
2016–2017	4913	11,782	40,487	38,818
2015–2016	4339	10,779	42,828	39,797
2014–2015	3298	8827	42,073	38,344
2013–2014	2714	7557	39,633	37,392
2012–2013	2027	6035	37,954	37,203
2011–2012	1761	5336	36,007	37,230
2010–2011	1300	4549	34,313	36,228
2009–2010	940	3498	31,842	34,134

*[((“personalized medicine”[All Fields]) AND (“20XX/10/22”[PDat]: “20XX + 1/10/21”[PDat])) OR ((“precision medicine”[All Fields]) AND (“20XX/10/22”[PDat]: “20XX + 1/10/21”[PDat]))], where 20XX is time interval from 2009 to 2021

**[(“medicine” [MeSH]) AND (“20XX/10/22”[PDat]: “20XX + 1/10/21”[PDat])], where 20XX is time interval from 2009 to 2021

### Parameters

The article identifiers (PMIDs) occurring in both samples were excluded from consideration, i.e., PMIDs were only used further in this study if they were uniquely identified as either target or control sample. The MeSH terms associated with five or fewer papers were removed from consideration to reduce the influence of rarely used terms and increase the reliability of the revealed differences between articles in the samples. The hierarchy of MeSH terms comprises 16 top-level categories (Mao & Lu, 2017). We limited our analysis to MeSH terms within the following three categories (and subcategories), namely: “Diseases” (C), “Chemicals and Drugs” (D), and “Analytical, Diagnostic and Therapeutic Techniques, and Equipment” (E).

At this stage of the analysis, we did not include papers from 2021–2022 in our study due to them being unrepresentative as a result of the lag in the MeSH terms assignment; the annotation of articles takes, on average, from 25 days to a year (Irwin & Rackham, 2017). About 10% of the articles remain uncharacterized concerning MeSH terms, despite their date of publication. The indexing time of a paper is, on average, 177 ± 100 days, 111 ± 69 days, and 23 ± 40 days for articles published in journals with impact factors of 2.0–2.5, 4.5–6.5, and > 25, respectively (Irwin & Rackham, 2017). The topic of the article also affects the indexing time. Articles in low-impact journals and non-English articles may remain unindexed.

### Technical requirements and algorithm limitations

To compare the frequency of the term in target and control samples, we use the proportion test. The more the number of tests (i.e. articles), the power of the criterion, i.e. more likely to find differences. For example, there is no significant difference in the frequencies 9/10 and 7/10, but if we increase the sample size (comparing already 90/100 and 70/100), then now this difference will already be significant. To compare frequencies, we use the prop.test function, which is based on calculating the chi-square statistic. The rule of thumb states that each number in the table must not be less than five, so we do not consider such MeSH terms that are assigned to less than 5 control and 5 target articles.

In a more common sense our algorithm focuses only on PubMed data. Most the biomedical articles are indexed in PubMed, but to apply our algorithm to other science fields, one should find the source of data. The results may be affected by the accuracy of keywords usage.

## Results

**Fig. 2:**
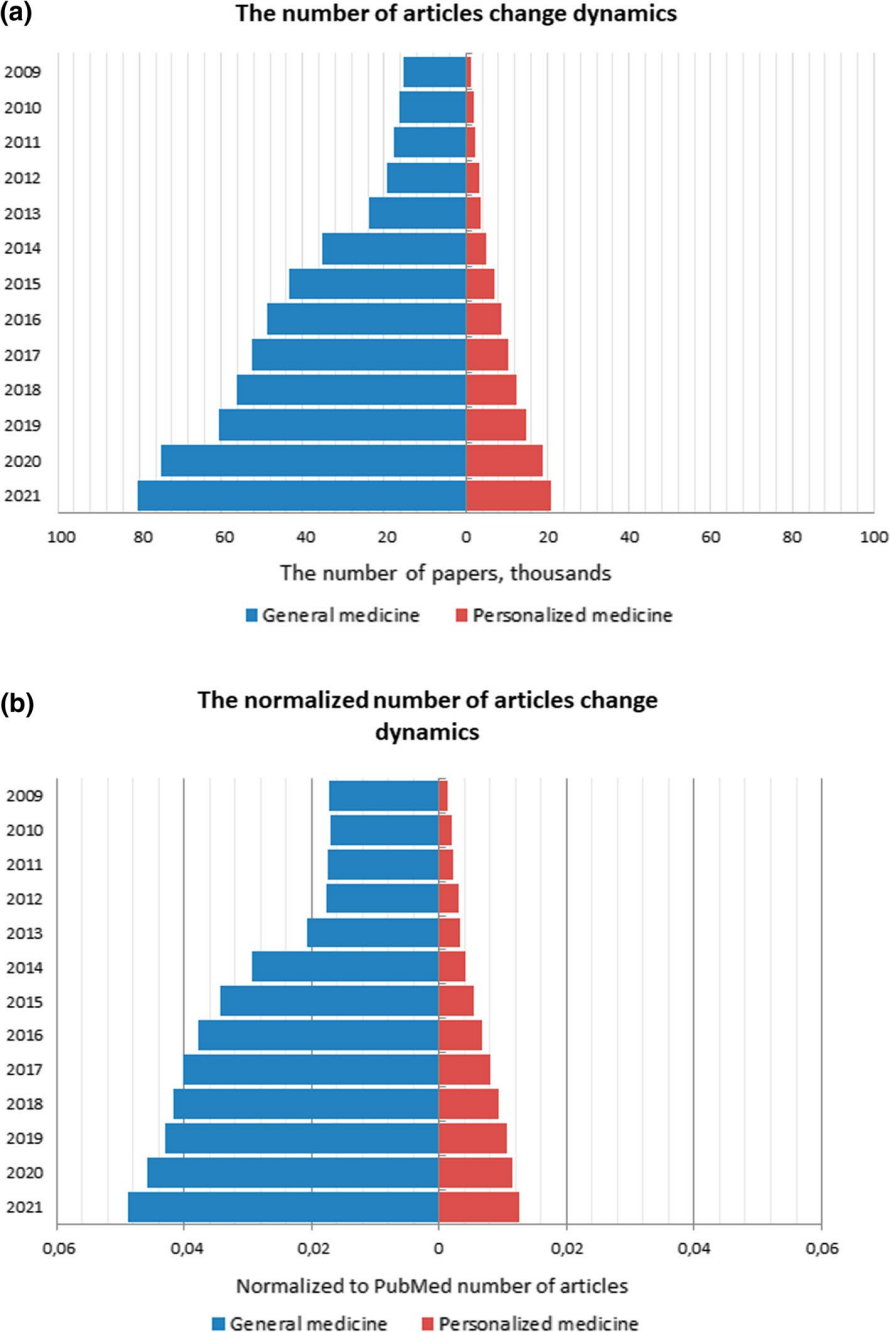
**a** Dynamics of changes in the number of articles on PubMed over the past decade and **b** the value normalized to the total number of articles in PubMed for articles. Pyramid on the left represents the increase in articles in control samples, while the pyramid on the right represents the increase in target samples. Normalization was done by dividing the control samples and target samples values by the absolute values of the articles from PubMed

The Fig. 2 reflects a natural increase in articles in target and control samples over the past decade, which correlates with an absolute increase in the number of articles on PubMed as a whole. Indeed, in the past decade alone, the number of articles has increased by one order of magnitude—from hundreds of thousands to over a million articles per year. This is due to the increased availability of data, improvements in experimental methods, and the acceptance of a concept in which articles are a measure of a scientist's effectiveness.

It was found that the number of unique MeSH terms is significantly correlated (*R*^2^ = 0.98) with the number of articles in the sample. On average, each article adds 2.8 ± 0.5 MeSH terms to the collection of unique terms.

The number of specific MeSH terms for the PM-articles is almost twice the number of terms specific to other articles in the field of medicine, while the total number of GM-articles exceeds the size of the target sample by 5 times. The relative diversity of terms may be associated with a relatively new field for which the established terminology is not yet characteristic. On the other hand, taking into account the vagueness of the very concept of “personalized medicine”, this can be considered as a consequence of a lack of clarity regarding which studies may actually belong to this field. Interestingly, while 12 years later, there was an increase in articles on the subject of personalized medicine by 4 times (from 3% in 2009 to 4% in 2021) of the total number of articles on medical topics. This indicates that a gradual translation of genomic and postgenomic scientific research into the field of practical medicine has occurred. A steadily decreasing number of MeSH terms are becoming specific for PM, which leads to the convergence of general and personalized medicine.

**Fig. 3:**
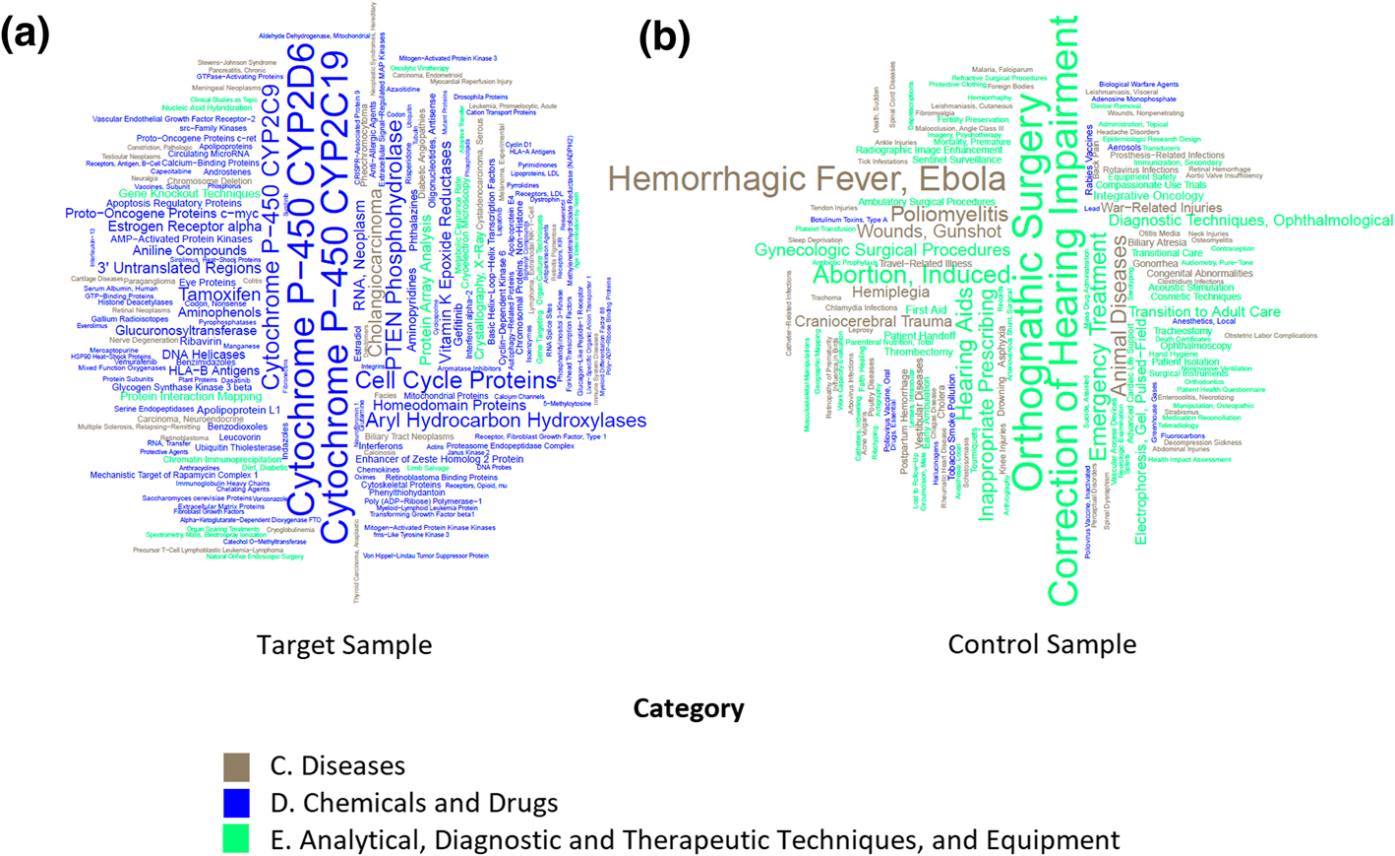
MeSH terms specific to articles relative to **a** “personalized and precision medicine” query and **b** “Medicine” [MeSH] in the PubMed/MEDLINE library for the period 2009–2021. Diseases (**C**) category is gray, chemicals and drugs (**D**) is blue, and Analytical, Diagnostic and Therapeutic Techniques and Equipment (**E**) is green. The font size is proportional to the frequency of the occurrence of the MeSH term

Each of the analyzed samples was converted into a set of characteristics—MeSH terms. The Fig. 3 shows an example of the distribution of terms specific to each sample (See materials and methods) in wordcloud format.

This way of presenting data makes it possible to quickly visualize the key differences in the analyzed directions. So, in the example under consideration, it is clear that personalized medicine related to the category Chemicals and Drugs: names of the drugs, e.g., Tamoxifen (antitumor) and Ribavirin (antiviral), as well as the names of enzymes from the cytochrome P-450 group involved in the metabolism of xenobiotics (CYP2D6, 2C19, 2C9). A substantial portion of the terms reflects the molecular processes of the pathogenesis of diseases, reflecting a significant contribution of biochemistry and molecular biology to the development of personalized medicine. There are relatively few keywords reflecting specific research methods in the field of personalized medicine (marked in green in Fig. 3), including experimental (“gene knockout techniques”, “crystallography X-ray”), and bioinformatics methods (“protein interaction mapping”).

In the trends of medicine (Fig. 3b), the situation is the opposite. The field belongs to the Analytical, Diagnostic and Therapeutic Techniques and Equipment category with a few MeSH terms from the Diseases category and even fewer from Chemicals and Drugs category. Keywords characterizing articles not related to personalized medicine can be considered as “negative” examples, that is, directions that are farthest from personalized medicine. According to the MeSH pattern presented in such terms (Fig. 3b), these directions include hearing problems (“correction of hearing impairment”, “hearing aids”), surgery (“orthognathic surgery”, “gynecologic surgical procedures”), public health problems (“inappropriate prescribing”, “transition to adult care”, “emergency treatment”) and the veterinary medicine (“animal diseases”). It can be seen that among the over 10 million articles in the PubMed/MEDLINE library, there are no studies linking the concepts of acute viral disease (“hemorrhagic fever, Ebola”) with personalized medicine.

Figure 3 once again confirms that the focus of personalized medicine is an individual person whose molecular portrait is captured by modern technologies with various details, while the medicine that is opposite to personalized is focused on population studies, for example, in the context of epidemic research and diseases of a viral nature. This is confirmed by the keywords “poliomyelitis”, “epidemiologic research design”, “geographic mapping”, and even “biological warfare agents”, a term that began to appear in papers in recent years.

**Fig. 4:**
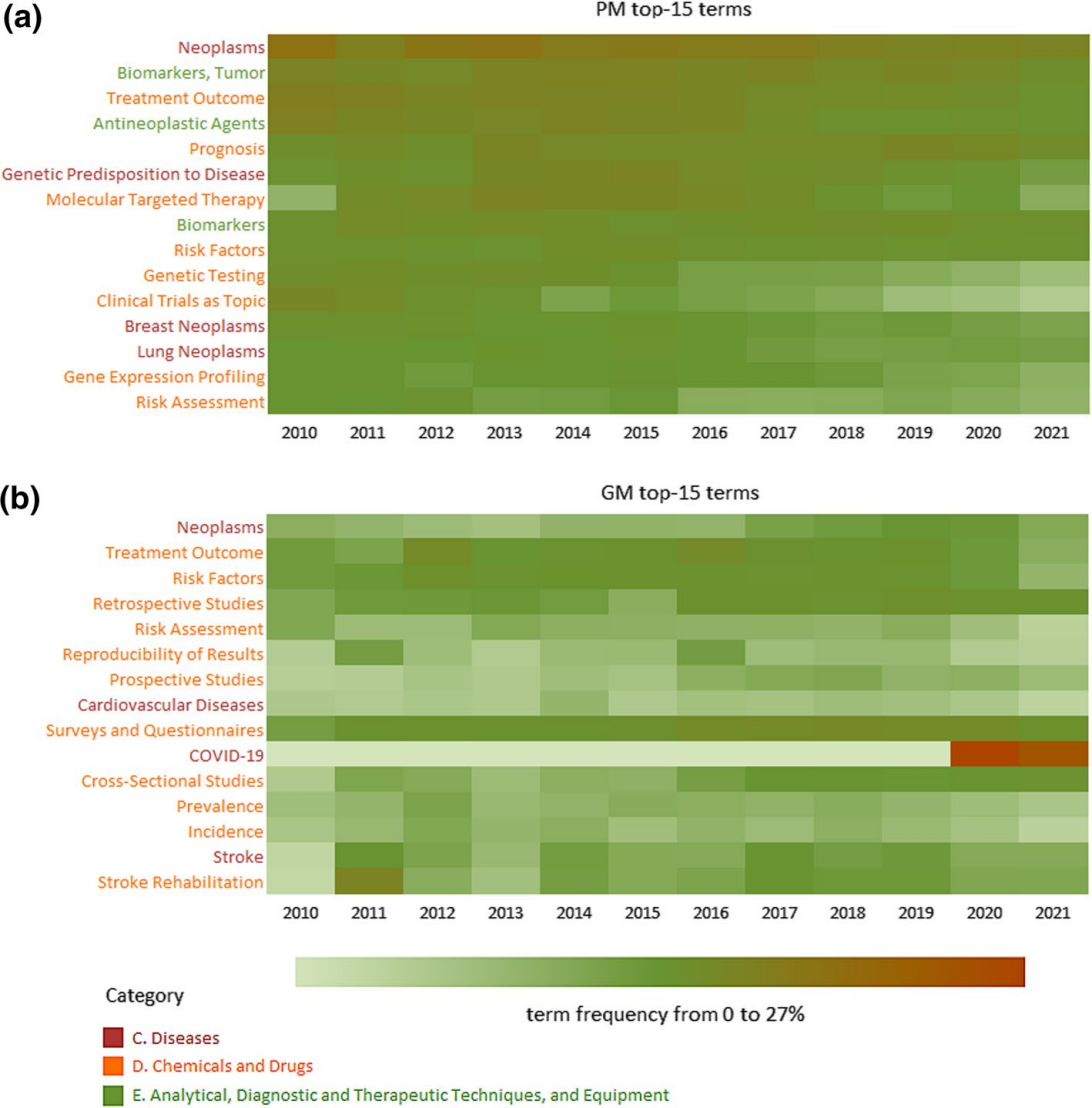
A fragment of a heatmap showing the proportions of papers indexed by the most common MeSH terms in a sample of papers in the field of **a** personalized medicine and **b** other medical papers

MeSH terms, which characterize articles in one or another analyzed sample, were compared in terms of frequency of occurrence. Among the most characteristic terms for each sample, we analyzed the changes in the frequency of occurrence in the period 2010–2021 to assess the relevance and significance of studies associated with a particular Mesh-term. The relative frequency of occurrence of MeSH terms was calculated for each interval (1 year), comparing the proportion of articles indexed by each particular MeSH term in the samples. Data on the frequency of occurrence of terms are given on Fig. 4 and in Supplementary Tables S3 and S4. The results are presented in the form of a heatmap reflecting the share of publications indexed by a given MeSH term in a given sample in a certain period. It can be seen that the more intense the coloring, the greater the proportion of articles indexed by this term, and vice versa. A general heatmap for 959 terms is given in Supplementary Note. Data on every Mesh term can help one to follow highly specialized trends.

As example, the term COVID-19 stands out significantly. First appearing in 2020, it reached 27% of the frequency, which is almost 8 times higher than the average values for other terms. These values allowed the term to enter the top-15 most common medicine-associated MeSH-terms in 2 years. In the field of personalized medicine, the term became widespread, but its indicators were noticeably lower. From the above, we can conclude that, as mentioned earlier, epidemics largely characterize the field of general medicine, setting its main trends. Indeed, with the spread of the infection, it becomes difficult to move from general to specific approaches and to treat patients in a personalized manner. There is simply not enough time and resources for personalized medicine.

Most of the MeSH-terms in Fig. 4 refer to medicine articles that characterize the descriptions of classical methods of conducting a medical examination when working with large groups of patients: studies (“surveys and questionnaires”, “prospective and retrospective studies”, “cross-sectional”), risk assessment, and risk factors. On the contrary, the "PM" articles are characterized by keywords related to the use of multi-omics approaches for “digitizing” the state of an individual patient in comparison with a digital portrait of a population: “genetic testing”, “gene expression profiling”, and “genetic predisposition to disease”. The group of keywords describing diseases includes “breast neoplasms” and “lung neoplasms” among the most important pathological conditions investigated in personalized medicine. The differences between the target and control samples approaches can be seen in the fact that the highest-ranked 15 terms of the control sample include the term “reproducibility of results”, since classical medical approaches are based on the “statistical” dogma of evidence-based medicine, and personalized medicine is “N-of-1 size” (Kolker et al., 2016).

Figure 4 shows that, depending on the ratio of the frequencies between the samples and the frequency distribution of the keyword over time, the analyzed keywords can be used to characterize the directions of studies in the field.

I.e., studies with the tags “neoplasms” and “biomarkers” characterize directions of research in which there are hardly any significant changes and “spikes” in interest; most likely, in the next 5 years, research in these fields will continue in the same direction and the predicted share of papers will be comparable to the currently determined shares.

A number of terms are characterized by an increase in the share of papers and, consequently, research interest (i.e., “molecular targeted therapy”, “immunotherapy”, “DNA mutational analysis”, “survival analysis”, “cohort studies”, “retrospective studies”, and more than 4 times over 10 years—“critical care”,”hemorrhage”). For some terms, on the contrary, a decrease (from 2 to 4 times) in the number of studies over the past 10 years (i.e., “drug delivery systems”, “drug monitoring”, “drug-related side effects and adverse reactions”, “models, biological”, “molecular diagnostic techniques”, “genetic markers”, “alcoholism”, and more than 4 times—“oligonucleotide array sequence analysis”, “drug therapy”, “Ras proteins”, “recombinant proteins”).

The format for data presentation proposed in our study allows a quick comparison of the trends found for two different directions. For example, the presented stable monotonous trend for “neoplasms” is not specific to personalized medicine (the target sample): other fields of medicine (the control sample) also show a consistently high proportion of publications with this tag (see Fig. 3b). Other similar examples are articles indexed by the terms “prognosis” and “treatment outcome”. A consequence of the increased interest in “cohort studies” in medicine (the proportion of articles indexed by this term in the control sample is growing) is the emergence of this term among the trends in personalized medicine, although this term characterizes an approach that is the opposite of “personalization”. Undoubtedly, when solving the problem of choosing the current direction in the field, it is necessary to take into account the dynamics of changes in the frequency of occurrence of key terms in recent years in the control and target samples.

Based on evaluations of *p*-value (whereby the Mann–Kendall test indicated it did not exceed 0.01) for 959 analyzed MeSH terms, those whose frequency of occurrence had significantly changed over 10 years (2010–2021) were selected. A total of 47 such terms were found; that is, about 5% of the total number of terms were considered “trends" in the field of personalized medicine (target sample). As a comparison, only 12 such terms were found in the field of medical papers (control sample) not related to personalized medicine. This observation once again confirms that personalized medicine is less “conservative” and stable compared to other medical areas, for the description of which there is a well-established set of basic concepts that are reflected in the articles, and their frequency remains more or less constant.

**Fig. 5:**
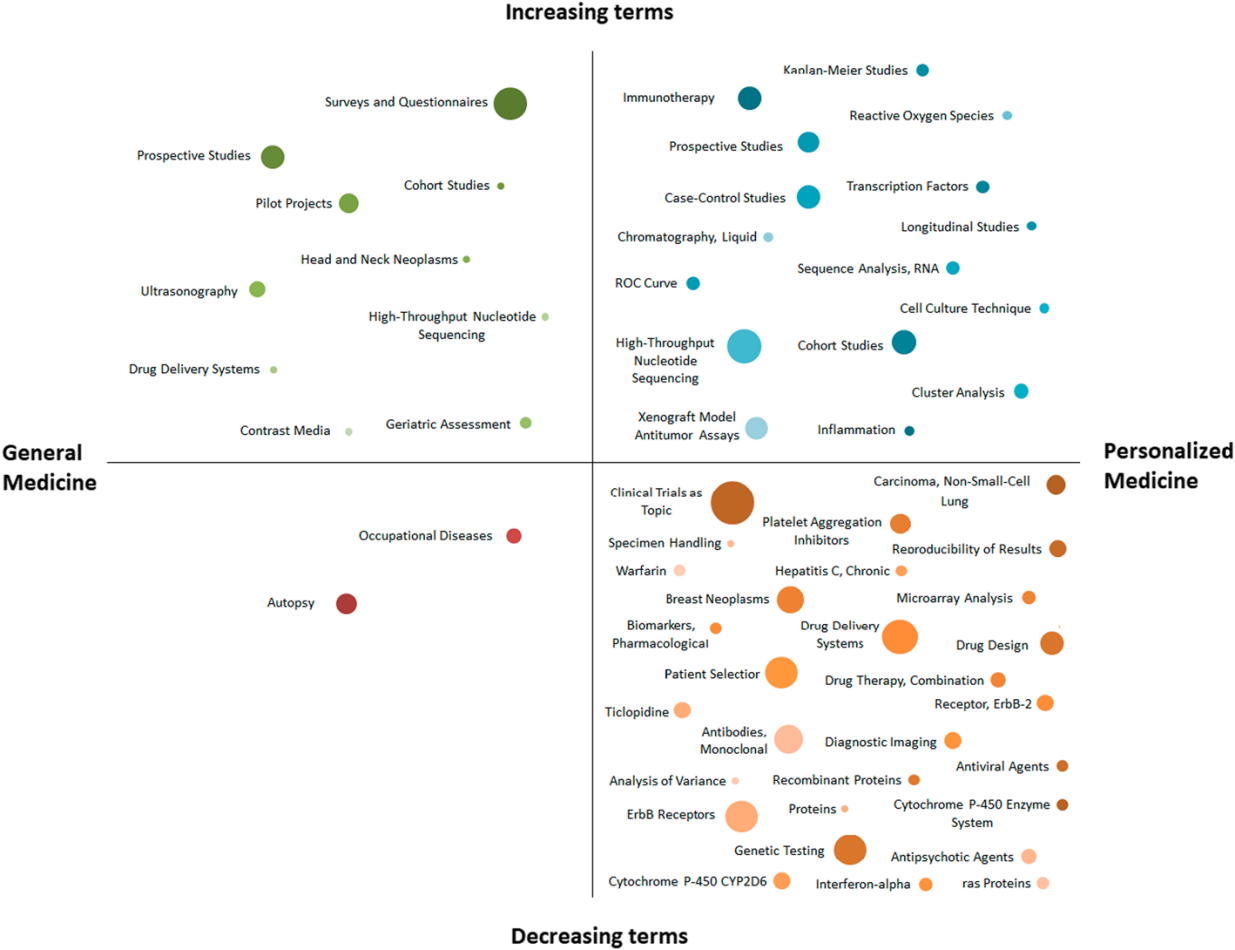
Dynamics of the frequency of use of terms in control and target samples. At the top are terms whose frequency of use is significantly decreasing, while the bottom indicates terms whose frequency of use in personalized medicine is significantly increasing. The reliability of the trend was determined using the Mann–Kendall test. The size of the dots reflects the degree of frequency change

A comparison of the reliable trends presented in Fig. 5 shows that the number of downward trends exceeds the number of upward trends. Thus, in the field of personalized medicine, an increase in the frequency of use of terms is observed only in 35% of cases, and the percentage of articles indexed by other terms of a given list is steadily decreasing. The opposite trend is characteristic of other articles in the field of medicine: the number of keywords characterizing negative trends is significantly less than the number of upward trends.

It is notable that the largest negative trend was identified for the term “precision medicine”. In 2008, 79% of articles returned from using PM as a query were annotated with this MeSH term; by contrast, in 2018, the share of such articles fell to 45%. It is important to mention that the terms “precision medicine and “personalized medicine” are united under the same MeSH term. One possible explanation may be that in the early 2000s, authors focused more on the phenomenon of personalized/precision medicine and, then, in later publications, the emphasis shifted to details (various instrumental methods, drugs, diseases, etc.) that is also reflected in the spectrum of MeSH terms.

A significant decrease in the relative frequency of use for PM articles was also shown for the terms “clinical trials as topic” (− 5.2%), “treatment outcome” (− 4.1%), “drug delivery systems” (− 3.5%), and “patient selection” (− 2.9%) (see Fig. 5). Based on the analysis of articles that are not related to the field of personalized medicine, a significant decreasing trend was found in the frequency of use for the terms “autopsy” (− 1.1%) and “occupational diseases” (− 0.7%).

It is of note that among some directions that characterize negative trends in the field of personalized medicine (i.e., “drug delivery systems”), similar keywords characterize the growth of interest in other non-personalized fields of medicine. Thus, the term “drug delivery systems” characterizes an upward trend based on the analysis of a GM sample of papers. In addition, increased interest is observed in the topics “surveys and questionnaires” (+ 4%). Other terms that showed a significant increase in relative frequency of use for GM articles are “prospective studies” (+ 1.9%), “pilot projects” (+ 1.5%), “ultrasonography” (+ 0.9%), “geriatric assessment and high-throughput nucleotide sequencing”. The latter term is also included in the list of upward trends in the field of personalized medicine (see Fig. 4a), however, in contrast to the relatively monotonous growth of most other trends, this term is characterized by a decrease starting from 2017. This decrease was also noted in the data for 2021, which is probably due to the fact that high-throughput sequencing, in itself, has become relatively routine in clinical practice. Other terms that characterize fields of study with an upward trend are “cohort studies” (+ 1.9%), “case–control studies” (+ 1.7%), “immunotherapy” (+ 1.6%), and “xenograft model antitumor assays” (+ 1.5%). These terms characterize the current directions of studies in the field of personalized medicine at the present time.

## Conclusions

Here we propose an approach to biomedicine trends analysis based on the comparison of two or more sets of papers, describing control and target research fields with the frequency of MeSH terms occurrence. Using the example of personalized and general medicine data sets, it is shown that there are keywords that characterize “persistent hot topics”, that is, areas of stable and strong interest (i.e., “neoplasms”, “treatment outcome”, “antineoplastic agents”); directions of downward trends (“oligonucleotide array sequence analysis”, “drug therapy”, “Ras proteins”, “recombinant proteins”); and those that are characterized by upward trends (“critical care”, “hemorrhage”). All the obtained results were mapped to other papers in the field of medicine in order to separate the trends that are specific rather than characteristic of medicine in general. Thus, among the upward trends in general medicine, studies in the field of “survey and questionnaires” can be distinguished.

Analysis of dynamics of more than 900 MeSH terms obtained over the past 10 years enables one to determine the areas attracting research attention in addition to predicting whether there will be an increase or decrease in interest for a particular direction over a three- to five-year period.

## Supplementary Information

Below is the link to the electronic supplementary material.

Supplementary file1 (PDF 617 kb)

Supplementary file2 (XLSX 242 kb)

## Data Availability

The data and materials used in the article are available in Supplementary Materials.
