# Supplementary figures and images for: How to catch trends using MeSH terms analysis?
Source: Scientometrics. 2022 Feb 21;127(4):1953–67. doi: 10.1007/s11192-022-04292-y (PMC8859845; doi:10.1007/s11192-022-04292-y)

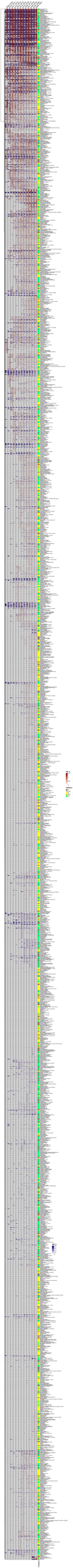

Supplement: Supplementary file 1 — Supplementary file1 (PDF 617 kb) [file 11192_2022_4292_MOESM1_ESM.pdf]
